# Bilateral Maxillary, Sphenoid Sinuses and Lumbosacral Spinal Cord Extramedullary Relapse of CML Following Allogeneic Stem Cell Transplant

**Published:** 2016-04-01

**Authors:** Soudabeh Hosseini, Shahla Ansari, Parvaneh Vosough, Gholamreza Bahoush, Amir Ali Hamidieh, Bahram Chahardouli, Morteza Shamsizadeh, Mitra Mehrazma, Akbar Dorgalaleh

**Affiliations:** 1 Department of Hematology and Blood Transfusion, School of Allied Medical Sciences, Iran University of Medical Sciences, Tehran, Iran; 2 Hematology-Oncology and Stem Cell Transplantation Research Center, Tehran University of Medical Sciences, Tehran, Iran; 3 Department of Medical-Surgical Nursing, School of Nursing and Midwifery, Hamadan University of Medical Sciences, Hamadan, Iran; 4 Department of Pathology, Iran University of Medical Sciences, Tehran, Iran

**Keywords:** Granulocytic sarcoma, Chronic myelogenous leukemia, Allogeneic stem cell transplantation

## Abstract

Isolated extramedullary relapse of chronic myelogenous leukemia (CML) after allogeneic stem cell transplant is rare. There is a case report of a child who developed a granulocytic sarcoma of the maxillary and sphenoid sinuses and lumbosacral spinal cord mass 18 months after allogeneic bone marrow transplant for CML. He was presented with per orbital edema and neurological deficit of lower extremities and a mass lesion was found on spinal cord imaging. No evidence of hematologic relapse was identified at that time by bone marrow histology or cytogenetic. The patient died 1 month later with a picture of pneumonia, left ventricular dysfunction and a cardiopulmonary arrest on a presumed underlying sepsis with infectious etiology. Granulocytic sarcoma should be considered in the differential diagnosis of mass lesions presenting after allogeneic bone marrow transplantation for CML, even if there is no evidence of bone marrow involvement.

## Introduction

Granulocytic sarcomas or as they were previously known chloroma are solid extra medullary masses composed of the blast forms of myeloid neoplasms. They could be manifestations of acute myelogenous leukemia (AML) or chronic myelogenous leukemia (CML) in blast crisis. They are very rare as an isolated presentation of relapse after allogeneic stem cell transplant (SCT), particularly in patients with CML.^1^^,^^2^

We report here the case of a child who developed a granulocytic sarcoma of the maxillary, sphenoid sinuses and lumbosacral spinal cord as an isolated manifestation of CML relapse 18 months after allogeneic SCT.

## Case report

An 18 month-old boy presented in January 1999/03 with leukocytosis and a white blood cell count of: 54400/mm^3^, RBC: 2.27 M/mm3, HGB: 9.1 g/dL, HCT: 26.1 %, MCV: 115 fL, MCH: 40.1 pg, MCHC: 34.9 g/dL, Platelet: 475000 K/mm3.

Bone marrow aspiration and biopsy revealed granulocytic hyperplasia with an increased M/E ratio (20-30/1), basophilia and increased neutrophiles. Differential diagnosis considered at that time was leukomoid reaction and chronic myeloid leukemia.

The immunophenotyping analysis by flowcytometry was as follows:

CD2 (0.4%), CD3 (12%), CD4 (0.1%), CD5 (2%), CD7 (4%), CD10 (0.1%), CD13 (25%), CD14 (2%), CD15 (60%), CD19 (1%), CD20 (1%), HLADR (12%), CD45 (80%), CD34 (2%), CD117 (1%) which was consistent with an increased mature myeloid component and could be presumptive of CML or a reactive leukomoid reaction.

These two entities could be differentiated by cytogenetic study of Philadelphia chromosome or BCR-ABL fusion gene detection. In a cytogenetic study of bone marrow despite absence of the Philadelphia chromosome and normal 46 XY karyotype, a positive BCR-ABL P210 fusion gene was detected by RT-PCR technique which was consistent with CML diagnosis.

The patient was initially treated with hydroxy urea, ARAC and INF-α till 2005 while still in chronic phase the WBC count raised up and imatinib was started. Initially, there was a very good response to imatinib but due to the side effect of severe headache, imatinib was discontinued and he was again treated with ARAC, INF-α and hydroxy urea.

Patient was under control till 2007/12, later because of another rise in WBC count imatinib was started again with a good response but it was always with severe arthritis (without warmness and redness) persisted in both elbows and right knee joint. On sonography of affected joints, effusion was detected along with an unbearable pain. Due to the increased ESR with the suspicion to septic arthritis, it was decided to perform an arthrotomy procedure on affected joints. Synovial fluid had fluidity with decreased viscosity. Bacterial culture of this fluid was negative. No evidence of septic arthritis was observed but empiric antibiotic therapy was continued. Severe arthritis pain due to administration of imatinib was relieved after 2 weeks following drug discontinuation. On 2007/12/02, while the patient was still in chronic phase, due to the side effects of imatinib, he was considered as a good candidate for allogenic peripheral blood SCT from his HLA- matched sibling donor. There had been a good response along with a slight graft versus host disease (GVHD) reaction which he received cyclosporine to control its side effects.

On 2009/5, bone marrow biopsy, bone marrow aspiration and touch imprints showed megaloblastic changes in erythroid and myeloid cells. Megakaryocytic cells were normal in number and morphology, granulocytic component also was normal, while erythroid and monocytic components of bone marrow were increased.

On 2009/6, analysis of STR of whole blood sample showed 65-70% chimerism. He received 2 DLI and later on short tandem repeat analysis of whole blood sample he had a 45-50% of chimerism. On 2009/6, gimsa banding cytogenetic study showed 46 XY t(9; 22) (q34; q11) + der(22) /46/XY corresponding with Philadelphia chromosome and an abnormal male karyotype accompanied by a clone of diploidy.^3^

On 2009/7, the result of analysis for T3151 mutation by RT PCR from Shariati Hospital laboratory was positive. On 2009/8, he was put on nilotinib. During this period, he always had periorbital edema and headache. On 2010/3, in brain CT scan, bilateral maxillary and sphenoid sinus mucosal thickening were observed. He also developed another complication on his lower extremities to the extent that he was not able to walk. On 2010/6, pelvic MRI showed enhancing soft tissue in presacral space in favor of tumoral mass and lumbosacral spine. MRI also showed inferior thecal sac enhancement in sacral canal in favor of malignancy. Due to recurrent sinusitis, periorbital edema on MRI technique and severe headache, sinusitis was diagnosed. On 2010/7, he went under sinus surgery with the probability of periorbital cellulites or fungal sinusitis. On 2010/7, paranasal biopsy showed respiratory type mucous infiltrated by rather monotonous large cells with high N/C ratio indented nucleolus with inconspicuous nucleoli which was very much consistent with an extramedullary malignant tumor (chloroma). On 2010/7/10, BM aspiration revealed > 60% blasts, megakaryocytes normal in number and morphology, orderly erythroid maturation along with megaloblastic changes. Moreover, bone marrow trephine biopsy showed crushed bone trabeculla, 80% cellularity and monomorphic population of hematopoietic elements. No megakaryocytes suggestive of CML blastic crisis were noted.

Evaluation of bone marrow biopsy myeloperoxidase in Shariati Hospital laboratory showed MPO (50%) positivity and CD 34 (90%) positivity on blast like cells. IHC was diagnostic for blastic crisis of CML. On 2010/8/19, he had cellulitis on left eye without tenderness and his chest x-ray on 2010/8/25 showed cardiomegaly.

On 2011/1, cytogenetic study revealed a 46 XY t(9; 22) (q34; q11), del(10) (q21) /46, XY/ idem, add(2) (q23) compatible with Ph chromosome.^4^

Sixty to eighty percent of CML cases develop secondary chromosomal aberration (CAs) during progression of disease which is usually reflective of the progressive nature of the disease. Peripheral blood lymphocyte karyotyping (PBLs) was also recommended to rule out the possibility of constitutional chromosome aberrations.

On 89/4/12, he was again admitted to the hospital with severe headache and sinusitis. He had an upward gaze and epileptic seizure and metastatic meningitis was considered as a differential diagnosis but on normal CSF cytology and brain CT scan, this probability was almost ruled out.

On 2010/7/3, on his last admission to Aliasghar Hospital, he had respiratory distress and tachycardia. O2 saturation was between 70-80% and he had a cardiopulmonary arrest due to left ventricular dysfunction.

## Discussion

 Leukemia-specific transcripts may be detected in the blood and bone marrow of individual CML patients for some months following allogeneic stem cell transplantation^5^^,^^6^^,^^7^ and their level may predict the risk of relapse.^8^^,^^4^ it was showed in one study that patients with no BCR-ABL transcripts detected between 3 and 5 months after transplantation had a probability of relapse at 3 years of 16.7%, whereas patients with low level and relatively high transcript levels had risks of relapse of 42.9% and 86%, respectively.^4^ When patients were categorized at 5 years after transplantation on the basis of prior transcript pattern, the subsequent risk of relapse was related to the transcript pattern in the interval preceding the 5-year point.^9^ However, the study revealed that some patients had transcripts detectable at low level for long periods after SCT without obvious progression. Because the clinical significance of finding these transcripts at low level was not known, therefore a criteria was proposed for molecular relapse (MR) designed to exclude patients in whom transcripts were present only at low levels, regardless of how long such low levels might persist.^9^

A chloroma is an extramedullary manifestation of acute myeloid leukemia; in other words, it is a solid collection of leukemic cells occurring outside of the bone marrow. Chloromas are rare; exact estimates of their prevalence are lacking but they are uncommonly seen even by physicians specializing in the treatment of leukemia. In general, chloromas must be regarded as an early herald of a systemic relapse, rather than as a localized process. In one review of 24 patients who developed isolated chloromas after treatment for acute myeloid leukemia, the mean interval until bone marrow relapse was 7 months (range: 1 - 19 months).

Although extramedullary manifestations of relapse following allogeneic SCT for myeloid malignancies are not unusual, occurring in up to 20% of patients transplanted for AML,^10^ but granulocytic sarcomas presenting as an isolated manifestation of relapse of either AML or CML after allogeneic SCT are rare. In one retrospective multicenter survey, only 0.45% of 5284 patients transplanted for myeloid malignancies (AML, CML or myelodysplastic syndrome) developed isolated granulocytic sarcomas.^1^Granulocytic sarcomas were more frequent in patients transplanted for AML than in patients with CML or MDS (0.65% vs. 0.22%). In a retrospective analysis of a single center experience, only 5 patients presented with isolated granulocytic sarcomas out of 134 individuals transplanted for CML or AML.^2^ Only 2 of these patients had CML; both presented with sacral lesions (this report excluded isolated skin or CNS recurrences). Only a few other cases of isolated extramedullary relapse in patients treated with allogeneic SCT for CML have been reported. ^11^^-^^15^ Myeloid sarcomas occurred in a variety of locations including the spine, dural space and spinal nerve roots, lymph nodes, clavicle, orbit, skin and pericardium. Granulocytic sarcomas involving the sphenoid and maxillary sinus have not been reported previously in the post-transplant patients. Although the small number of patients reported make it difficult to draw conclusions, it has been hypothesized that the occurrence of granulocytic sarcomas in the absence of medullary relapse of CML is more likely in the absence of GVHD, due to a lack of graft-versus leukemia effect.^2^ The presence of a stronger graft-versus- leukemia effect in bone marrow than in peripheral tissues has also been proposed.^12^

Various treatment approaches have been used for isolated granulocytic sarcomas in the post-transplant CML patient. Long-term outcomes have been reported for 12 patients. Two had long-term survival: one after local radiation alone^1^ and another after local radiation followed by a second allogeneic SCT.^2^ Four patients have been reported dead with hematologic relapse after receiving radiation and unspecified chemotherapy.^1^ Two patients achieved remission after donor lymphocyte infusion died at 3 months (one of GVHD and infection, one of GVHD and CNS relapse).^11^ One patient died of progressive local disease despite radiation and steroids.^2^ Three more patients have been reported dead within several months of infections after initially responding to radiation alone or (in one case) combined with cytarabine and thioguanine.^12^^,^^13^

Donor lymphocyte infusion (DLI) is less effective in acute leukemia and other hematologic tumors. The appropriate role of DLI for post transplant relapse is still being defined. The high rate of durable complete remissions in CML has been determined. One could argue that DLI is the treatment of choice in relapsed CML. But in our patient in spite of two DLIs, appropriate response was not observed and the improper trend of laboratory and clinical manifestations was continued. The toxicity of DLI can be substantial and some relapsed CML patients may benefit from interferon-α therapy.

Observations in humans have suggested strongly the existence of a GVL effect in human allogeneic BMT. Relapse rates in syngeneic BMT recipients are higher than in allogeneic BMT recipients. Allogeneic BMT recipients who do not develop GVHD relapse more frequently than allogeneic BMT recipients who do develop GVHD; and recipients of T-cell-depleted BMT relapse more frequently than recipients of non-T-cell-depleted BMT. In addition, complete remissions have been observed in patients with relapsed disease after allogeneic BMT in association with flares of GVHD.

Based on the apparent power of the GVL effect and its presumed mediation by donor leukocytes (T cells and/ or natural killer (NK) cells), leukocytes obtained from the original bone marrow donor have been infused into patients with relapsed malignancy after allogeneic BMT. Durable complete remissions have been reported in 50% to 70% of patients with chronic myelogenous leukemia (CML). Sporadic responses have also been observed in patients with other relapsed hematologic malignancies after allogeneic BMT.

For better understanding of benefits and toxicities of DLI, relapsed patients after allogeneic HSCT should be assessed. Recipients of allogeneic BMTs who have relapsed may attain complete remissions when treated with transfusions of leukocytes obtained from the original bone marrow donor. The best therapy for patients with relapse after SCT remains to be defined, especially with the advent of imatinib as a very active drug in the treatment of chronic myelogenous leukemia (CML).

## CONCLUSION

 Sarcoma should be a part of the differential diagnosis of patients who present with mass lesions after allogeneic transplant for CML, even in the absence of hematologic relapse. Imatinib mesylate is a small molecule tyrosine kinase inhibitor that has significant efficacy in the treatment of CML. However, it is likely that patients with CML will require prolonged and perhaps life-long therapy. In general, the side-effects of imatinib therapy have been mild to moderate, with the large majority of patients tolerating prolonged periods of therapy. However, a minority of patients are completely intolerant of therapy, while others are able to remain on therapy despite significant side-effects.

Here, we described second case of fluid retention presenting as multiple joint effusions in a patient with advanced phase CML on high-dose imatinib. Although fluid retention including periorbital edema, pleural and pericardial effusions, as well as life-threatening cerebral edema has been previously described and attributed to imatinib, this is the second case of imatinib-associated polyarticular effusions. The first case was reported by Moore JC et al.^16^ Further work will be required to confirm a casual relationship between imatinib therapy and this novel side-effect, as well as to determine the underlying pathophysiologic mechanisms for this side effect.

Our patient while still in chronic phase underwent an allogeneic peripheral blood SCT from his HLA-antigen matched sibling donor but bone marrow transplantation has better outcome if performed early in the course of the disease before treatment abandonment.
